# AUF1 Restrains Hepatocyte Senescence by Maintaining Mitochondrial Homeostasis in AML12 Hepatocyte Model

**DOI:** 10.3390/cells15010048

**Published:** 2025-12-26

**Authors:** Myeongwoo Jung, Sukyoung Han, Seungyeon Ryu, Seongho Cha, Ye Eun Sim, Se Hoon Jung, Hyosun Tak, Wook Kim, Eun Kyung Lee

**Affiliations:** 1 Department of Biochemistry, College of Medicine, The Catholic University of Korea, Seoul 06591, Republic of Korea; 2 Institute for Aging and Metabolic Diseases, College of Medicine, The Catholic University of Korea, Seoul 06591, Republic of Korea; 3 Department of Medical Science, Graduate School of The Catholic University of Korea, Seoul 06591, Republic of Korea; 4 Cancer Research Center of Lyon (CRCL), UMR INSERM U1052/CNRS 5286, 69008 Lyon, France; 5 Department of Molecular Science & Technology, Ajou University, Suwon 16499, Republic of Korea; 6 Advanced College of Bio-Convergence Engineering, Ajou University, Suwon 16499, Republic of Korea

**Keywords:** senescence, mitochondria, AUF1, fusion, reactive oxygen species

## Abstract

Cellular senescence, a hallmark of aging, involves irreversible growth arrest and an enhanced senescence-associated secretory phenotype (SASP). It is often accompanied by mitochondrial dysfunction and altered inter-organelle communication. Using a chronic oxidative stress model in AML12 hepatocytes, we confirmed senescence by canonical assays (e.g., SA β-gal positivity and proliferation arrest) and observed a decline in the RNA-binding protein AUF1 (hnRNP D). AUF1 knockdown further amplified senescent phenotypes, including elongation of mitochondrial network, loss of mitochondrial membrane potential, reduced ATP level, and elevated mitochondrial reactive oxygen species (ROS). In addition, AUF1 knockdown weakened mitochondria-endoplasmic reticulum coupling and reduced mitochondrial Ca^2+^ load. At the molecular level, AUF1 binds to the 3′ untranslated regions (3′UTRs) of *Opa1* and *Mfn2* and limits their abundance, thereby coupling post-transcriptional control to mitochondrial dynamics. In gain-of-function experiments, ectopic expression of AUF1 attenuated Opa1/Mfn2 induction, restored mitochondrial network architecture, and preserved bioenergetic function under pro-senescent stimuli. Collectively, these findings support a model in which AUF1 preserves mitochondrial homeostasis and thereby restrains the mitochondria–senescence axis in hepatocytes.

## 1. Introduction

Cellular senescence is a stress-responsive state characterized by growth arrest, chromatin remodeling, mitochondrial dysfunction, and the acquisition of senescence-associated secretory phenotype (SASP) [1,2,3]. Several studies have shown that mitochondrial dysfunction is not merely a byproduct but a central driver of cellular senescence; impaired quality control and imbalanced fission–fusion elevate reactive oxygen species, release mitochondrial DNA, and rewire bioenergetic signaling. These changes, in turn, reinforce growth arrest and prime pro-inflammatory pathways that ultimately feed into the SASP [3,4]. Senescent cells, therefore, do not simply withdraw from the cell cycle; rather, they actively reshape the surrounding microenvironment and modulate the behavior of neighboring immune and stromal cells by secreting pro-inflammatory cytokines, chemokines, growth factors, and matrix-remodeling enzymes [5,6,7]. Because the SASP can propagate senescence-like changes in a paracrine manner and perpetuate low-grade inflammation, it is increasingly regarded as a therapeutic target rather than a passive correlate of aging [1,2,5,8,9]. The liver is particularly susceptible to paracrine and immunological cues due to its rich repertoire of non-parenchymal cells and continuous exposure to blood-borne signals. This suggests that senescence in hepatocytes may readily translate into organ-level consequences rather than affecting other tissues [10,11,12,13].

Hepatocyte senescence is increasingly recognized as a driver of chronic liver injury and metabolic dysfunction-associated steatotic liver disease (MASLD), because senescent hepatocytes can transmit pro-fibrogenic and pro-inflammatory signals to non-parenchymal cells, promote hepatic stellate cell (HSC) activation and hepatic remodeling, and worsen steatotic inflammation [10,14]. Recent reports further suggest that activation of senescence programs in hepatocytes may influence systemic metabolic and inflammatory states, highlighting that hepatocyte senescence is not a passive correlate but a therapeutically actionable process [15]. However, the molecular mechanisms that drive its initiation and maintenance—and the key hepatocyte-intrinsic regulators that orchestrate these steps—remain poorly defined.

AUF1, also known as hnRNP D, is an RNA-binding protein that regulates mRNA stability and translation by binding to the 3′UTRs of target mRNAs, thereby modulating gene expression at the post-transcriptional level [16,17]. Alteration of AUF1 expression has been linked to the pathogenesis of several diseases, including inflammation, cancer, skin diseases, and muscle wasting diseases, and AUF1-deficient mice show hallmarks of premature aging [18,19,20,21,22]. In this study, we demonstrated that AUF1 expression is reduced in a senescent AML12 hepatocyte model. Functional depletion of AUF1 further enhanced the senescent phenotype of AML12 cells, as evidenced by an increase in senescence markers and enhanced mitochondrial dysfunction. Mechanistically, AUF1 knockdown upregulated the expression of mitochondrial fusion factors Opa1 and Mfn2, thereby disturbing the balance of mitochondrial dynamics that is typically observed during senescence. These data identify AUF1 as an important post-transcriptional factor that limits hepatocyte senescence through maintenance of mitochondrial dynamics and suggest that restoring AUF1 levels could represent a useful strategy for targeting senescent hepatocytes in metabolic liver disease.

## 2. Materials and Methods

### 2.1. Cell Culture, Transfection, and Viral Transduction

Mouse hepatocyte AML12 cells (American Type Culture Collection (ATCC), Manassas, VA, USA; CRL-2254) were purchased and cultured in Dulbecco’s Modified Eagle Medium/Nutrient Mixture F12 (DMEM/F12; Capricorn Scientific, Ebsdorfergrund, Germany) supplemented with 10% fetal bovine serum (FBS), 1% antibiotics, 1× Insulin-Transferrin-Selenium Pyruvate Supplement (ITSP; Welgene, Gyeongsan, Republic of Korea), and 100 nM dexamethasone (Sigma-Aldrich, Burlington, MA, USA) at 37 °C. Small interfering RNAs (siRNAs; BIONEER, Daejeon, Republic of Korea) and plasmids were transfected using Lipofectamine™ 2000 (Invitrogen™, Waltham, MA, USA), according to the manufacturer’s instructions. Sequences of siRNAs used in this study are provided in Supplementary Table S1. For adenoviral transduction, AML12 cells were infected with Ad-AUF1 virus (Ad-CMV-h-HNRNPD) or control adenovirus (Ad-CMV-Null) (Vector Biolabs, Malvern, PA, USA) at 2 × 10^8^ viral particles in serum-free medium for 4 h, after which the medium was replaced with complete culture medium.

### 2.2. Establishment of Senescent Hepatocyte Model

To establish a hepatocyte senescence model, AML12 cells were continuously exposed to H_2_O_2_ for 7 days, following the protocols described in [23]. Briefly, on Day 1, cells were treated with 1 mM H_2_O_2_ (Sigma-Aldrich) in serum-free medium for 1 h at 37 °C. On Days 2–3, cells were treated with 750 μM H_2_O_2_ for 1 h each day at 37 °C and were subsequently sub-cultured on Day 3. On Days 4–6, cells were similarly treated with 750 μM H_2_O_2_ for 1 h daily at 37 °C, and cells were used for experiments on Day 7. Senescence induction was assessed by cell counting, BrdU incorporation assay (Merck, Taufkirchen, Germany), and TUNEL assays (Promega Corporation, Madison, WI, USA).

### 2.3. Immunoblotting Analysis

Cells were lysed using RIPA buffer (Biosesang, Inc., Seongnam, Republic of Korea) containing 1× protease inhibitor cocktail (Roche, Basel, Switzerland), and proteins were separated by SDS-PAGE. Proteins were transferred onto PVDF membrane (Millipore, Burlington, MA, USA) and incubated with primary antibodies against AUF1 (Merck; 07-260), p16 (Santa Cruz Biotechnology, Inc., Dallas, TX, USA; SC-1661), p21 (BD Bioscience, Franklin Lakes, NJ, USA; 556431), OPA1 (612606), DRP1 (611112), MFN2 (Abcam Plc., Cambridge, UK; ab56889), MFN1 (ab104274), MFF (ab81127), and β-actin (Genetex, Inc., Irvine, CA, USA; GTX629630), then sequentially incubated with HRP-conjugated secondary antibodies (Merck; AP124P and AP132P). Chemiluminescence was generated by Clarity Western ECL Substrate (Bio-Rad, Inc., Hercules, CA, USA) and detected by the ChemiDoc Imaging Systems (Bio-Rad, Inc.).

### 2.4. RNA Analysis and Ribonucleoprotein Immunoprecipitation (RNP-IP)

Total RNAs were extracted using RNAiso Plus (Takara Bio, Inc., Shiga, Japan) and reverse-transcribed into cDNA using the ReverTra^®^ Ace qPCR RT Kit (Toyobo Co., Ltd., Osaka, Japan). Relative RNA levels were analyzed by quantitative PCR (qPCR) using SensiFAST™ SYBR Hi-ROX kit (Meridian Bioscience, Inc., Cincinnati, OH, USA). Primer sequences used for qPCR are listed in Supplementary Table S1. Data were relatively quantified using the _ΔΔ_CT method, and *Gapdh* mRNA was used as the reference gene for normalization.

For RNP-IP, RNP complexes were immunoprecipitated using AUF1 (Merck; 07-260) or control IgG (Merck; 12-370) antibodies conjugated to Pierce™ Protein A Agarose (Thermo Fisher Scientific, Waltham, MA, USA). RNA was isolated from the RNP complexes, reverse-transcribed into cDNA, and analyzed using qPCR as described previously [24,25].

### 2.5. Biotin Pulldown Assay

For synthesis of biotinylated RNA probes, PCR fragments containing T7 RNA polymerase binding sequence ([T7]:CCAAGCTTCTAATACGACTCACTATAGGGAGA) were amplified using Blend Taq^TM^ (Toyobo Co., Ltd.) and specific primers targeting the 3′UTR of mouse *Opa1* (NM_001199177) and *Mfn2* (NM_001285920) listed in Supplementary Table S1. The amplified PCR products were transcribed in vitro using MaxiScript T7 kit (Ambion, Waltham, MA, USA) and biotin-CTP (Enzo Life Sciences, Farmingdale, NY, USA). The biotinylated RNA probes were incubated with cell lysates and pulled down using Dynabeads™ Streptavidin Magnetic Beads (Invitrogen™), and the binding of AUF1 to RNAs was assessed by immunoblotting.

### 2.6. Fluorescence and Electron Microscopy Analysis

For immunofluorescence microscopy, cells were fixed using 4% FA (Biosesang, Inc.) and permeabilized with 0.4% Triton X-100 in PBS. After blocking, cells were incubated with primary antibody against NDUFV2 (Proteintech Group, Inc., Rosemont, IL, USA; 15301-1-AP) and further incubated with secondary antibody conjugated with Alexa Fluor^®^ 555 (Abcam Plc.; ab150074). Fluorescence signals from mitochondria were imaged using a ZEISS LSM 900 confocal microscope (Carl Zeiss, Oberkochen, Germany). Mitochondrial number, area, perimeter, and branch length were analyzed using the Mitochondrial Analyzer plugin (https://github.com/AhsenChaudhry/Mitochondria-Analyzer, accessed on 23 December 2025) for ImageJ/Fiji software version 1.54p (NIH, Bethesda, MD, USA), as previously described methods [26].

For analysis of mitochondrial Ca^2+^ levels and mitochondria-ER contact sites (MERCs), cells were transfected with pcDNA-4mtD3cpv (mitochondrial Ca^2+^ sensor; Addgene, Watertown, MA, USA; #36324) [27], SPLICS Mt-ER Short P2A (Addgene #164108), and SPLICS Mt-ER Long P2A (Addgene; #164107) [28,29]. Fluorescence signals from fixed cells were acquired using a ZEISS Axio Imager Z1 microscope (Carl Zeiss) and quantified using ImageJ/Fiji software (NIH) [30,31].

For transmission electron microscopy (TEM), cells were fixed with 2.5% glutaraldehyde and embedded in epoxy resin (Polysciences, Inc., Warrington, PA, USA). Ultrathin sections were prepared using an ultramicrotome (Leica Microsystems Ltd., Wetzlar, Germany), and mitochondrial morphology was examined using an HT7800 transmission electron microscope (Hitachi, Tokyo, Japan).

### 2.7. Analysis of Mitochondrial ATP Level and Membrane Potential

Mitochondrial ATP levels and membrane potential (Δψ_m_) were analyzed using the Mitochondrial ToxGlo™ Assay (Promega Corporation; G8000) and the JC-1 Mitochondrial Membrane Potential Assay Kit (Abcam Plc.; ab113850), respectively, according to the manufacturer’s instructions. Luminescence signals from ToxGlo™ Assay and fluorescence signals from JC-1 (Ex/Em = 530/590 nm) were measured using a BioTek Synergy H1 microplate reader (Agilent Technologies, Winooski, VT, USA).

### 2.8. Assessment of Senescence Markers

Cell proliferation was evaluated by cell counting using a hemocytometer, and cell morphology was examined using a Leica DM IL LED microscope (Leica Microsystems Ltd.). For senescence-associated β-galactosidase (SA β-gal) staining, cells were incubated with an SA β-gal staining solution (40 mM citric acid/sodium phosphate buffer, pH 6.0; 150 mM NaCl; 2 mM MgCl_2_; 5 mM potassium ferricyanide; 5 mM potassium ferrocyanide; and 1 mg/mL X-gal (BEAMS Biotechnology, Seongnam, Republic of Korea) at 37 °C for 16 h in the dark, and stained cells were subsequently examined using an Olympus IX70 microscope (Olympus Corp., Tokyo, Japan). SA β-gal-positive cells were quantified using ImageJ/Fiji software. The expression of senescence markers was assessed by RT-qPCR and immunoblotting analyses.

### 2.9. Measurement of Reactive Oxygen Species (ROS)

Intracellular and mitochondrial ROS levels were analyzed using 5 μM CM-H_2_DCFDA (Invitrogen™) and 5 μM MitoSOX™ Red mitochondrial superoxide indicator (Invitrogen™), respectively, according to the manufacturer’s instructions. CM-H_2_DCFDA fluorescence intensity was evaluated using a FACS Canto™ flow cytometer (BD Biosciences). MitoSOX™ fluorescence signals were visualized using a ZEISS LSM 900 confocal microscope (Carl Zeiss) and quantified with ImageJ/Fiji software.

### 2.10. Statistical Analysis

Data are represented as the mean  ±  SEM of three independent experiments. Statistical significance was determined using Student’s *t*-test for comparisons between two groups and one-way ANOVA with Tukey’s test for multiple comparisons. (* *p* < 0.05; ** *p*  <  0.01; *** *p*  <  0.001).

## 3. Results

### 3.1. AUF1 Is Downregulated in a Senescent AML12 Hepatocyte Model Induced by Chronic Oxidative Stress

**Figure 1:**
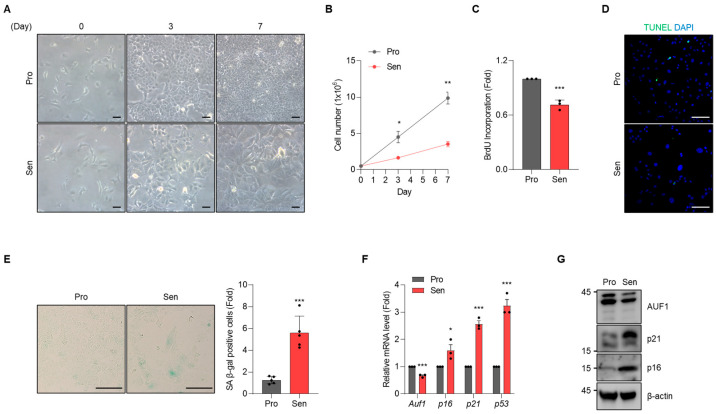
Chronic oxidative stress induces senescence in AML12 hepatocytes and downregulates AUF1. A senescent hepatocyte model was established using AML12 cells as described in [23]. Cell morphology (**A**) and number (**B**) were assessed on Days 0, 3, and 7. BrdU incorporation assay (**C**) and TUNEL assay (**D**) were used to determine cell proliferation and cell death. (**E**) SA β-gal staining. SA β-gal-positive cells were quantified by cell counting. (**F**) RT-qPCR. *Gapdh* mRNA was used for normalization. (**G**) Immunoblotting analysis. β-actin, a loading control. Pro, proliferating; Sen, senescent. Scale bar, 100 μm. Representative images from three independent biological replicates (*n* = 3). * *p*  <  0.05; ** *p*  <  0.01; *** *p*  <  0.001.

To investigate hepatocyte-intrinsic mechanisms of cellular senescence, we established a senescent AML12 model by continuously exposing the cells to H_2_O_2_ following the STAR Protocols method [23]. Over the time course, cells developed characteristic morphological changes (Figure 1A), accompanied by a progressive decrease in cell number (Figure 1B), which appeared to reflect reduced proliferation rather than increased cell death (Figure 1C,D). By Day 7, senescence was confirmed by an increased portion of SA β-gal-positive cells (Figure 1E) and elevated expression of canonical markers, including *p16*, *p21*, and *p53* (Figure 1F). Levels of p21 and p16 were increased in senescent cells (Sen) compared with proliferating control cells (Pro) (Figure 1G). These results indicate that cellular senescence was successfully induced in AML12 cells. Notably, AUF1 expression was significantly reduced at both mRNA and protein levels in senescent AML12 cells compared to proliferating controls (Figure 1F,G), suggesting that AUF1 may act as a regulator of hepatocyte senescence.

### 3.2. Senescent AML12 Cells Exhibit Fusion-Dominant Mitochondrial Remodeling, Bioenergetic Impairment, and MERCs Reorganization

**Figure 2:**
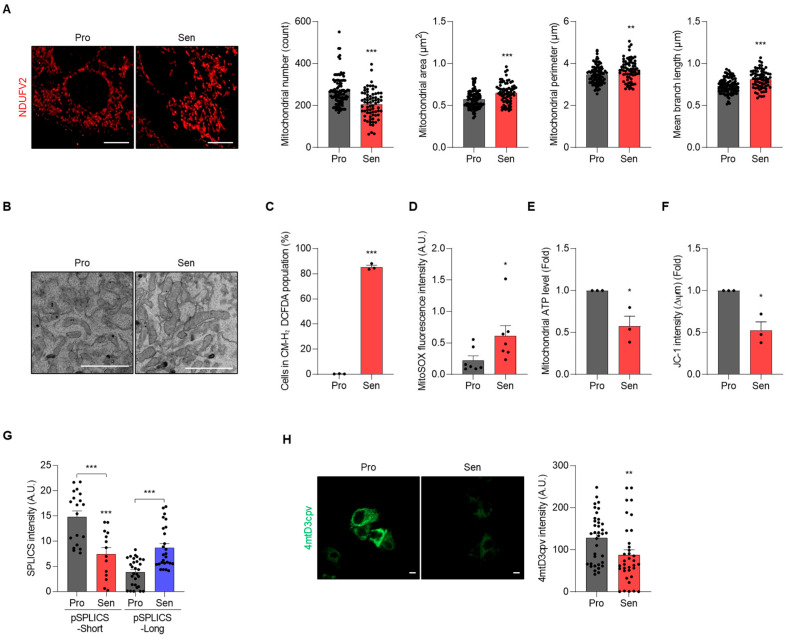
Senescent AML12 hepatocytes show mitochondrial network elongation, MERCs reorganization, and bioenergetic impairment. (**A**) Mitochondrial morphology was analyzed by immunofluorescence microscopy using the NDUFV2 antibody. Mitochondrial number, area, perimeter, and branch length were quantified using ImageJ/Fiji software. Each dot represents a mitochondrial measurement from an individual cell. (**B**) TEM images. (**C**) Cellular ROS levels (CM-H_2_DCFDA staining). (**D**) Mitochondrial ROS levels (MitoSOX™ staining). (**E**) Mitochondrial ATP levels (Mitochondrial ToxGlo™). (**F**) Mitochondrial membrane potential (JC-1 staining). (**G**,**H**) MERCs were monitored using the split-GFP contact-site sensor SPLICS system (**G**), and mitochondrial Ca^2+^ levels were determined using the mitochondria-targeted FRET probe 4mtD3cpv (**H**). Fluorescence of each probe was analyzed by ImageJ/Fiji software. Scale bar, 10 μm for (**A**,**H**), and 1 μm for (**B**). Representative images from three independent biological replicates (*n* = 3). * *p*  <  0.05; ** *p*  <  0.01; *** *p*  <  0.001.

Mitochondrial dysfunction is a hallmark of cellular senescence [3,32,33]. To investigate mitochondrial architecture and function in the senescent AML12 model, we assessed mitochondrial networks by quantitative morphometry following immunofluorescence staining for the complex I subunit NDUFV2 [26]. Relative to proliferating control cells, senescent AML12 cells exhibited a reduced mitochondrial number, accompanied by an increased mean area, perimeter, and branch length, consistent with an elongated, fusion-dominant morphology (Figure 2A). This structural remodeling was corroborated by TEM, which revealed more elongated mitochondria in senescent AML12 cells (Figure 2B). In line with chronic oxidative stress, cellular ROS (CM-H_2_DCFDA) and mitochondrial ROS (mitoSOX) were elevated in senescent cells (Figure 2C,D). Functionally, senescent AML12 cells showed reduced mitochondrial ATP synthesis and diminished mitochondrial membrane potential (Δψ_m_) as measured by JC-1 staining, indicating a bioenergetic compromise (Figure 2E,F).

Given the established function of mitochondria-endoplasmic reticulum contact sites (MERCs) in regulating Ca^2+^ exchange and thereby influencing mitochondrial dynamics and bioenergetics [34,35,36], we next asked whether MERCs are remodeled in senescent AML12 cells. Using the split-GFP contact-site sensor SPLICS [28,29], we monitored short-range (SPLICS-S) and long-range (SPLICS-L) mitochondria-ER appositions. As shown in Figure 2G, senescent AML12 cells exhibited reduced SPLICS-S fluorescence with a concomitant increase in SPLICS-L signal compared to proliferating control cells, indicating a loss of tight MERCs and a shift toward more distant contacts. Consistent with the reduced contacts, mitochondrial Ca^2+^ uptake, assessed with the mitochondria-targeted FRET probe 4mtD3cpv [27], was diminished in senescent cells (Figure 2H). Collectively, these data demonstrate that senescent AML12 cells undergo mitochondrial elongation accompanied by MERCs reorganization and impaired Ca^2+^ transfer, providing a mechanistic link to the observed bioenergetic decline.

### 3.3. AUF1 Knockdown Promotes a Senescent Phenotype in AML12 Hepatocytes

**Figure 3:**
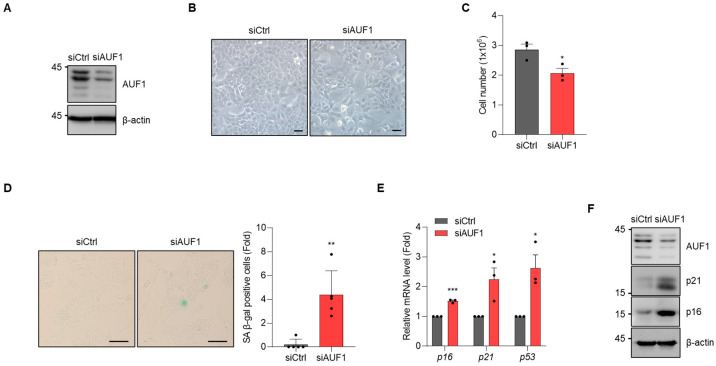
AUF1-KD promotes a senescent phenotype in AML12 hepatocytes. AML12 cells were transfected with siRNAs and cultured for 72 h. (**A**) AUF1 downregulation was confirmed by immunoblotting. (**B**) Cell morphology. (**C**) Cell number. (**D**) SA β-gal staining. SA β-gal-positive cells were quantified by cell counting. (**E**) RT-qPCR. *Gapdh* mRNA was used for normalization. (**F**) Immunoblot analysis. β-actin, a loading control. Scale bar, 100 μm. Representative images from three independent biological replicates (*n* = 3). * *p*  <  0.05; ** *p*  <  0.01; *** *p*  <  0.001.

To determine whether AUF1 is a functional regulator of hepatocyte senescence, we downregulated AUF1 in AML12 cells by transient transfection of siRNA and confirmed AUF1 downregulation at the protein level (Figure 3A). AUF1-knockdown (AUF1-KD) AML12 cells developed the characteristic senescent morphology, becoming larger and more flattened in shape (Figure 3B). Furthermore, AUF1-KD decreased cell number and increased the number of SA β-gal-positive cells in AML12 cells (Figure 3C,D). In addition, AUF1 downregulation increased the expression of senescence markers at both the mRNA and protein levels (Figure 3E,F). These results indicate that AUF1 reduction is sufficient to drive AML12 hepatocytes into a senescent state.

### 3.4. AUF1-KD Induces a Senescent-like Mitochondrial Phenotype in AML12 Hepatocytes

**Figure 4:**
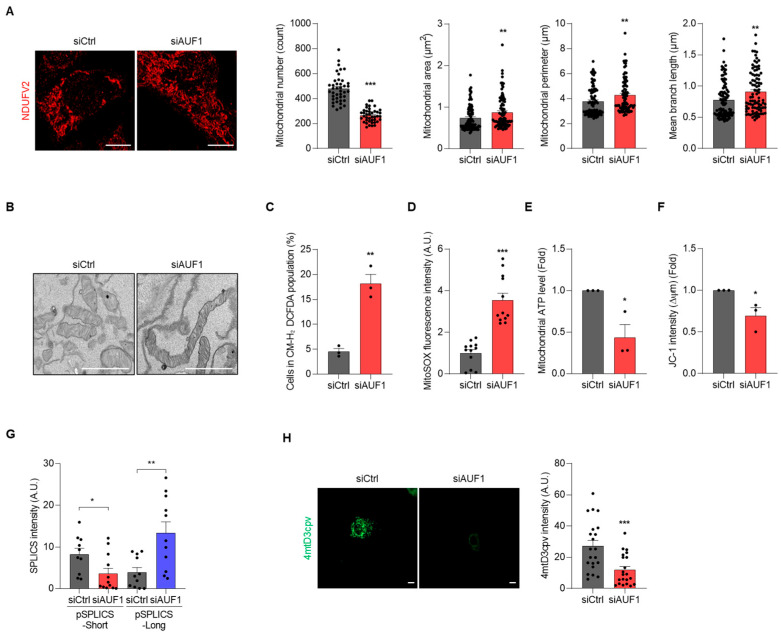
AUF1-KD results in senescence-like mitochondrial remodeling and reduces mitochondrial Ca^2+^ uptake. AML12 cells were transfected with siRNAs and cultured for 72 h. (**A**) Mitochondrial morphology was analyzed by NDUFV2 immunofluorescence. Mitochondrial number, area, perimeter, and branch length of each image were analyzed using ImageJ/Fiji software. Each dot represents a mitochondrial measurement from an individual cell. (**B**) TEM images. (**C**,**D**) Cellular ROS levels (CM-H_2_DCFDA staining) and mitochondrial ROS levels (MitoSOX™ staining). (**E**,**F**) Mitochondrial ATP levels (Mitochondrial ToxGlo™) and mitochondrial membrane potential (JC-1 staining). (**G**,**H**) MERCs (SPLICS) (**G**) and mitochondrial Ca^2+^ level (4mtD3cpv) (**H**). Fluorescence of each probe was analyzed by ImageJ/Fiji software. Scale bar, 10 μm for (**A**,**H**), and 1 μm for (**B**). Representative images from three independent biological replicates (*n* = 3). * *p*  <  0.05; ** *p*  <  0.01; *** *p*  <  0.001.

To determine whether AUF1 downregulation is sufficient to induce the mitochondrial abnormalities observed in senescent AML12 cells, we analyzed mitochondrial morphology, function, and MERCs organization under AUF1-KD. Analysis of NDUFV2 immunofluorescence and TEM revealed enhanced mitochondrial elongation in AUF1-KD cells (Figure 4A,B). ROS levels increased at both the whole-cell and mitochondrial levels relative to controls (Figure 4C,D). AUF1-KD also reduced mitochondrial ATP levels and decreased mitochondrial membrane potential (Figure 4E,F). Furthermore, short-range contacts (SPLICS-S) decreased, while long-range contacts (SPLICS-L) increased under AUF1-KD (Figure 4G). In accordance with this looser coupling between mitochondria and ER, mitochondrial Ca^2+^ uptake, as measured by 4mtD3cpv probe, was reduced in AUF1-KD cells (Figure 4H). Collectively, AUF1-KD resulted in mitochondrial remodeling and functional deficits as seen in senescent AML12 cells. These results suggest that AUF1 is an upstream regulator in the mitochondria-senescence axis in AML12 hepatocytes.

### 3.5. AUF1 Binds Mitochondrial Fusion Factor mRNAs and Contributes to Fusion-Dominant Structure

**Figure 5:**
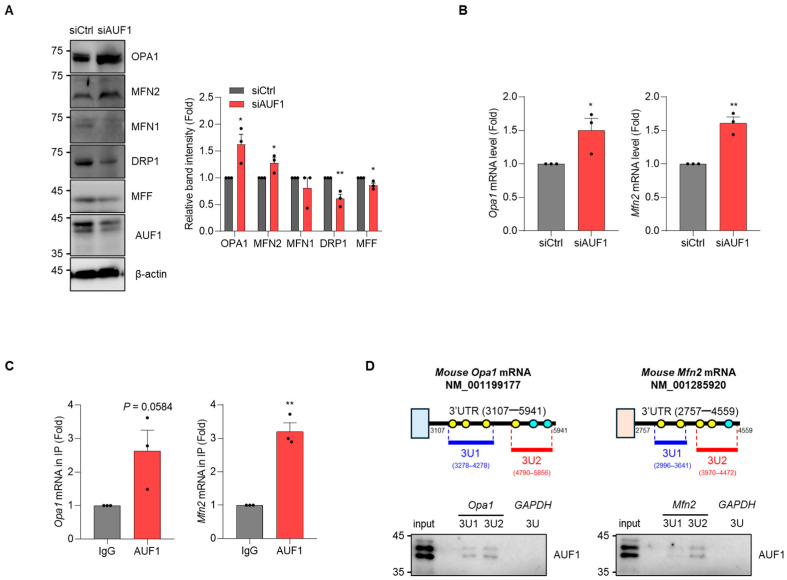
AUF1-KD increases mitochondrial fusion factors and binds their 3′UTRs. (**A**,**B**) After siRNA transfection of AML12, levels of proteins and mRNAs were determined by immunoblotting (**A**) and RT-qPCR (**B**), respectively. Immunoblotting images were quantified using Image Lab software (Version 6.1). (**C**) RNP-IP followed by RT-qPCR. AUF1-containing RNP complexes were isolated, and relative mRNA enrichment was assessed by RT-qPCR compared with IgG control. (**D**) (**Upper**) Schematic representation of the 3′UTR of mouse *Opa1* (NM_001199177) and *Mfn2* (NM_001285920) mRNA. RNA probes (3U1 and 3U2) containing the GU/UG-rich region (yellow) and AU-rich region (cyan) of each 3′UTR were synthesized. (**Lower**) The binding between each probe and AUF1 was assessed biotin pulldown assay followed by immunoblotting. *GAPDH* 3U was used as a negative control for the biotin pulldown assay. β-actin, a loading control for immunoblotting. *Gapdh* mRNA, a reference gene for normalization. Representative images from independent biological replicates (*n* = 3 for (**A**–**C**) and *n* = 2 for (**D**)). * *p*  <  0.05; ** *p*  <  0.01.

Because AUF1-KD induced a fusion-dominant mitochondrial morphology, we investigated whether AUF1 modulates the expression of genes governing mitochondrial dynamics. In AML12 hepatocytes, AUF1-KD increased expression of Opa1 and Mfn2, consistent with mitochondrial elongation; although Drp1 and Mff exhibited modest statistically significant changes, we focused subsequent analyses on Opa1 and Mfn2 to test a fusion-biased remodeling mechanism (Figure 5A). RT-qPCR confirmed increased levels of *Opa1* and *Mfn2* mRNAs in AUF1-KD AML12 cells (Figure 5B). Since AUF1 is an RNA-binding protein mainly linked to mRNA decay [19,22], we investigated whether *Opa1* and *Mfn2* mRNAs physically associate with AUF1. RNP-IP revealed enrichment of *Opa1* and *Mfn2* mRNAs in AUF1-containing ribonucleoprotein complexes compared with IgG control (Figure 5C). In silico analysis identified GU/UG-rich or AU-rich RNA sequences within the 3′UTRs of *Opa1* and *Mfn2* mRNAs that are predicted to be accessible for AUF1 (Supplementary Figure S1) [17,37]. A pulldown assay using biotinylated RNAs harboring defined 3′UTR fragments revealed direct interaction of AUF1 (Figure 5D). Taken together, these data suggest that AUF1 binds the mRNAs of *Opa1* and *Mfn2*, and limits their abundance. Loss of AUF1 therefore promotes expression of mitochondrial fusion factors, including Opa1 and Mfn2, shifting dynamics toward elongation and providing a mechanistic link between AUF1 deficiency and the mitochondrial phenotype-associated senescence.

### 3.6. AUF1-KD Exacerbates Mitochondrial Abnormalities and Accelerates Senescence Under Pro-Senescent Stress in AML12 Cells

**Figure 6:**
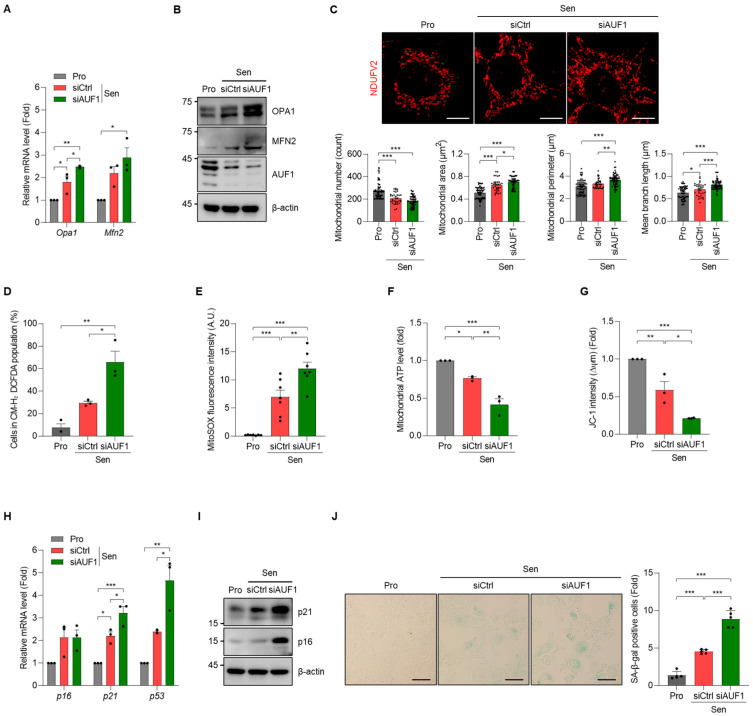
Senescence induction under AUF1-KD amplifies mitochondrial defects and senescence outputs. After siRNA transfection, senescence was induced in AML12 cells as described in Materials and Methods, and relative changes among proliferating control (Pro), senescent cells transfected with control siRNA (Sen-siCtrl), and senescent cells transfected with AUF1 siRNA (Sen-siAUF1) were analyzed. (**A**) Levels of *Opa1* and *Mfn2* mRNAs (RT-qPCR). (**B**) Levels of Opa1 and Mfn2 (immunoblotting). (**C**) Mitochondrial morphology analyzed by NDUFV2 immunofluorescence. Mitochondrial number, area, perimeter, and branch length were quantified using ImageJ/Fiji software. Each dot represents a mitochondrial measurement from an individual cell. (**D**,**E**) Cellular ROS levels (CM-H_2_DCFDA staining) and mitochondrial ROS levels (MitoSOX™ staining). (**F**,**G**) Mitochondrial ATP levels (Mitochondrial ToxGlo™) and mitochondrial membrane potential (JC-1 staining). (**H**) Levels of *p16*, *p21*, and *p53* mRNAs (RT-qPCR) (**I**) Levels of p16 and p21 (immunoblotting) (**J**) SA β-gal staining. SA β-gal-positive cells were analyzed by cell counting. β-actin, a loading control for immunoblotting. *Gapdh* mRNA, a reference gene for normalization. Representative images from three independent biological replicates (*n* = 3). Scale bar, 10 μm for (**C**), and 100 μm for (**J**). * *p*  <  0.05; ** *p*  <  0.01; *** *p*  <  0.001.

To investigate whether loss of AUF1 sensitizes hepatocytes to senescence-associated remodeling, we induced senescence using chronic oxidative stress (Figure 1) after AUF1-KD and then assessed mitochondrial dysfunction and expression of senescence markers (as in Figure 1 and Figure 2). Senescence induction further increased *Opa1* and *Mfn2* transcripts in AUF1-KD AML12 cells relative to control senescent cells (Sen-siCtrl), with parallel increases in Opa1 and Mfn2 proteins (Figure 6A,B). Quantitative morphometry likewise showed that senescence induction in AUF1-KD cells significantly increased mitochondrial perimeter, area, and branch length, consistent with accentuated network elongation (Figure 6C). Concomitantly, total ROS and mitochondrial ROS increased further, while mitochondrial ATP levels decreased and membrane potential became more depolarized compared to the control senescent AML12 cells (Figure 6D–G). At the cellular level, senescence markers, including *p16*, *p21*, and *p53*, exhibited higher levels after senescence induction (Figure 6H). Levels of p21 and p16 in senescent cells were further increased by AUF1-KD, and the population of SA β-gal-positive cells increased accordingly (Figure 6I,J). Together, these results suggest that AUF1 reduction primes hepatocytes for an amplified mitochondrial dysfunction and cellular senescence in response to pro-senescent stimuli, supporting a model in which AUF1 restrains the mitochondria–senescence axis.

### 3.7. Ectopic Expression of AUF1 Mitigates Mitochondrial Dysfunction and Stress-Induced Senescence in AML12 Cells

**Figure 7:**
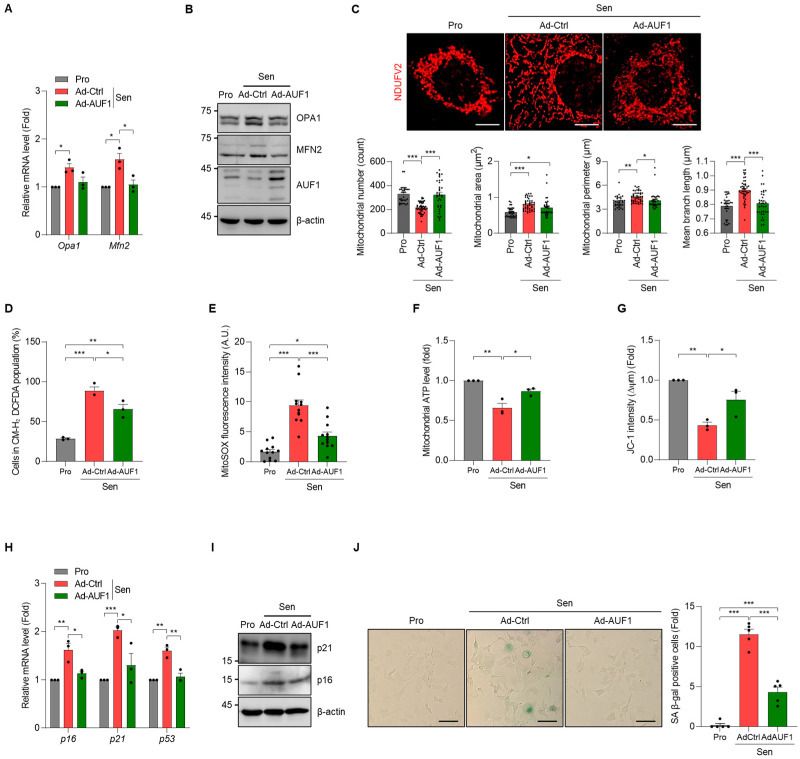
Ectopic expression of AUF1 preserves mitochondrial dynamics and attenuates senescence under pro-senescent stress. After adenoviral transduction, senescence was induced in AML12 cells, and relative changes among proliferating control (Pro), senescent cells transduced with control virus (Sen-Ad-Ctrl), and senescent cells transduced with AUF1 virus (Sen-Ad-AUF1) were analyzed. (**A**) Levels of *Opa1* and *Mfn2* mRNAs (RT-qPCR). (**B**) Levels of Opa1 and Mfn2 (immunoblotting). (**C**) Mitochondrial morphology was analyzed by NDUFV2 immunofluorescence. Mitochondrial number, area, perimeter, and branch length of each image were quantified using ImageJ/Fiji software. Each dot represents a mitochondrial measurement from an individual cell. (**D**,**E**) Cellular ROS levels (CM-H_2_DCFDA staining) and mitochondrial ROS levels (MitoSOX™ staining). (**F**,**G**) Mitochondrial ATP levels (Mitochondrial ToxGlo™) and mitochondrial membrane potential (JC-1 staining). (**H**) Levels of *p16*, *p21*, and *p53* mRNAs (RT-qPCR) (**I**) Levels of p16 and p21 (immunoblotting) (**J**) SA β-gal staining. SA β-gal-positive cells were analyzed by cell counting. β-actin, a loading control for immunoblotting. *Gapdh* mRNA, a reference gene for normalization. Representative images from three independent biological replicates (*n* = 3). Scale bar, 10 μm for (**C**), and 100 μm for (**J**). * *p*  <  0.05; ** *p*  <  0.01; *** *p*  <  0.001.

To evaluate whether AUF1 upregulation can counteract the mitochondrial remodeling and senescence-like phenotypes, we ectopically expressed AUF1 in AML12 cells, subjected the cells to senescence induction, and compared the effects of AUF1 rescue on mitochondrial dysfunction and cellular senescence, with proliferating control AML12 cells included as baseline controls. Induction of Opa1 and Mfn2 expression in senescent AML12 cells was attenuated by AUF1 expression (Figure 7A,B). Morphometric analysis of mitochondria showed that AUF1 expression retrieved the shift towards elongated networks upon senescence induction relative to vector-transduced senescent cells, consistent with maintenance of a more balanced mitochondrial architecture (Figure 7C). In addition, total ROS and mitochondrial ROS were lower, whereas mitochondrial ATP levels were higher and membrane potential better preserved in AUF1-expressing cells than in vector controls after senescence induction (Figure 7D–G). Levels of senescence markers were partially reduced compared with induced vector controls (Figure 7H,I), and the population of SA β-gal-positive cells was decreased by AUF1 expression (Figure 7J). Taken together with the loss-of-function data (Figure 3, Figure 4 and Figure 6), these gain-of-function results demonstrate that increasing AUF1 levels prevents fusion-dominant remodeling, bioenergetic decline, and elevation of senescence markers in response to pro-senescent stimuli. These findings support the notion that AUF1 is an essential regulator of the mitochondria-senescence axis in AML12 hepatocytes by maintaining mitochondrial homeostasis.

## 4. Discussion

This study identifies AUF1 as a hepatocyte-intrinsic regulator that restrains the mitochondria–senescence axis. Using a chronic oxidative-stress model in AML12 cells [23], we show that AUF1 is reduced in senescent hepatocytes, that AUF1 loss is sufficient to reproduce a fusion-biased mitochondrial state, bioenergetic decline, and amplified senescence readouts, and that ectopic AUF1 expression attenuates these changes. Mechanistically, AUF1 binds the 3′UTRs of *Opa1* and *Mfn2* mRNAs and limits their abundance, thereby helping to preserve the homeostasis of mitochondrial dynamics. Together, these findings position AUF1 as an essential checkpoint through which mitochondrial remodeling is coupled to hepatocyte senescence under conditions of chronic stress.

In this regard, the use of oxidative stress–based senescence models is well supported by in vivo evidence from chronic liver disease settings. Multiple mouse models of MASLD/NASH and liver fibrosis have demonstrated a sustained increase in hepatic ROS, accompanied by hepatocellular damage, and potential activation of senescence-associated pathways [38,39,40,41]. Across these models, genetic or pharmacological attenuation of ROS production, or enhancement of ROS clearance, significantly alleviates liver injury, inflammation, and fibrotic progression, underscoring a causal role of oxidative stress in disease pathogenesis [42]. Thus, the chronic oxidative stress–induced senescence model employed here recapitulates key pathological features observed in vivo and provides a disease-relevant yet experimentally tractable platform to interrogate hepatocyte-intrinsic regulators, such as AUF1, that link mitochondrial dysfunction to cellular senescence.

Our data indicate that AUF1 governs mitochondrial dynamics in the AML12 senescence model, and that AUF1 reduction is associated with loss of ΔΨ_m_, decreased ATP levels, and increased ROS, alongside enhanced mitochondrial fusion (Figure 2 and Figure 4). Complementing this hepatocyte-intrinsic role, recent work in human diploid fibroblasts showed that AUF1 modulates glycolytic flux by regulating *PDP2* and *PGAM1* mRNAs, thereby suppressing senescence [43]. Beyond mitochondria and metabolism, AUF1 has also been linked to core aging pathways; it has been reported to promote TERT expression and support telomere maintenance [44] and to influence chronic inflammatory output/SASP regulation [20,21,45,46]. Taken together, these lines of evidence highlight the importance of AUF1 in regulating cellular senescence. Nevertheless, the precise molecular circuitry and molecular mechanisms by which AUF1 integrates intracellular events remain to be fully resolved and warrant further study.

Despite the emerging importance of AUF1 in senescence, the regulation of AUF1 expression and activity in hepatocytes is poorly understood. Multiple regulatory layers are likely involved, including transcriptional inputs from stress-responsive factors that change AUF1 level under lipotoxic, inflammatory, or oxidative conditions [47]; post-transcriptional regulation mediated by microRNAs and RNA-binding proteins [48,49]; context-dependent composition and subcellular localization of AUF1 isoforms (p37/p40/p42/p45); and post-translational modifications that affect RNA binding affinity, protein stability, and intracellular localization [43]. Defining this upstream circuitry in hepatocytes, particularly in MASLD-relevant models, will be essential to determine whether AUF1 decline is causal or adaptive, clarify how it affects cellular senescence, and evaluate the feasibility of targeting AUF1 therapeutically.

The elongated mitochondrial networks that we observe in the senescent AML12 model (Figure 2) may reflect not only increased fusion but also reduced mitochondrial turnover [50,51]. In hepatocyte senescence models, autophagy has been reported to decline [52], raising the possibility that impaired mitophagy contributes to the accumulation of elongated, damaged mitochondria. To clarify the relative contributions of fusion bias versus diminished mitophagy flux, direct assays using specific probes, such as mt-Keima and COX8-mCherry-GFP, and time-resolved measurements during senescence induction would be informative. Establishing whether mitophagy is indeed reduced in senescent hepatocytes and how this intersects with AUF1-dependent remodeling is therefore a priority for follow-up work.

Taken together, our data suggest that AUF1 plays a pivotal role in AML12 hepatocytes by linking mitochondrial homeostasis to cellular senescence. More broadly, these findings suggest that aberrant expression of RNA-binding proteins can actively drive, rather than merely accompany, senescence. Important next steps include clarifying how AUF1 expression becomes downregulated in hepatocytes, delineating the breadth of its target mRNAs beyond Opa1/Mfn2, and defining whether AUF1 modulates mitophagy flux and organelle crosstalk (including MERCs architecture and Ca^2+^ handling). In addition, validation in primary hepatocytes and MASLD models is also required. If these avenues converge, AUF1 will emerge as a promising target for interventions in chronic inflammation and senescence-driven diseases, providing a potential approach to modifying liver pathology by restoring or regulating AUF1-dependent post-transcriptional control.

## 5. Conclusions

In summary, our study identifies AUF1 as a hepatocyte-intrinsic checkpoint that restrains the mitochondria–senescence axis. AUF1 loss promotes a fusion-biased mitochondrial remodeling program, accompanied by bioenergetic decline, increased mitochondrial ROS, and enhanced senescence-associated phenotypes. Mechanistically, AUF1 binds the 3′UTRs of *Opa1* and *Mfn2* mRNAs and limits their abundance. Conversely, restoring AUF1 expression attenuated these mitochondrial and senescence-associated changes, supporting a causal role for AUF1 in maintaining mitochondrial homeostasis under chronic stress. Together, these findings suggest that AUF1 contributes to modulating mitochondrial remodeling and cellular senescence.

## Data Availability

The data that support the findings of this study are available from the corresponding author upon reasonable request.

## Supplementary Materials

The following supporting information can be downloaded at https://www.mdpi.com/article/10.3390/cells15010048/s1: Figure S1: Analysis of AUF1 binding sequences in the 3′UTR of mouse *Opa1* and *Mfn2* mRNAs; Table S1: Oligo sequences used in this study.

## References

[1] Birch J., Gil J. (2020). Senescence and the SASP: Many therapeutic avenues. Genes Dev..

[2] Wang B., Han J., Elisseeff J.H., Demaria M. (2024). The senescence-associated secretory phenotype and its physiological and pathological implications. Nat. Rev. Mol. Cell Biol..

[3] Miwa S., Kashyap S., Chini E., von Zglinicki T. (2022). Mitochondrial dysfunction in cell senescence and aging. J. Clin. Investig..

[4] Bondy S.C. (2024). Mitochondrial Dysfunction as the Major Basis of Brain Aging. Biomolecules.

[5] Han Z., Wang K., Ding S., Zhang M. (2024). Cross-talk of inflammation and cellular senescence: A new insight into the occurrence and progression of osteoarthritis. Bone Res..

[6] Tchkonia T., Zhu Y., van Deursen J., Campisi J., Kirkland J.L. (2013). Cellular senescence and the senescent secretory phenotype: Therapeutic opportunities. J. Clin. Investig..

[7] Ohtani N. (2022). The roles and mechanisms of senescence-associated secretory phenotype (SASP): Can it be controlled by senolysis?. Inflamm. Regen..

[8] Li X., Li C., Zhang W., Wang Y., Qian P., Huang H. (2023). Inflammation and aging: Signaling pathways and intervention therapies. Signal Transduct. Target Ther..

[9] van de Vyver M. (2023). Immunology of chronic low-grade inflammation: Relationship with metabolic function. J. Endocrinol..

[10] Kiourtis C., Terradas-Terradas M., Gee L.M., May S., Georgakopoulou A., Collins A.L., O’Sullivan E.D., Baird D.P., Hassan M., Shaw R. (2024). Hepatocellular senescence induces multi-organ senescence and dysfunction via TGFβ. Nat. Cell Biol..

[11] Bonnet L., Alexandersson I., Baboota R.K., Kroon T., Oscarsson J., Smith U., Boucher J. (2022). Cellular senescence in hepatocytes contributes to metabolic disturbances in NASH. Front. Endocrinol..

[12] Jun J.H., Du K., Dutta R.K., Maeso-Diaz R., Oh S.H., Wang L., Gao G., Ferreira A., Hill J., Pullen S.S. (2024). The senescence-associated secretome of Hedgehog-deficient hepatocytes drives MASLD progression. J. Clin. Investig..

[13] Umbaugh D.S., Diehl A.M., Du K. (2025). Redefining senescence through hepatocyte fate changes in liver diseases. Trends Endocrinol. Metab..

[14] Wijayasiri P., Astbury S., Kaye P., Oakley F., Alexander G.J., Kendall T.J., Aravinthan A.D. (2022). Role of Hepatocyte Senescence in the Activation of Hepatic Stellate Cells and Liver Fibrosis Progression. Cells.

[15] Du K., Umbaugh D.S., Wang L., Jun J.H., Dutta R.K., Oh S.H., Ren N., Zhang Q., Ko D.C., Ferreira A. (2025). Author Correction: Targeting senescent hepatocytes for treatment of metabolic dysfunction-associated steatotic liver disease and multi-organ dysfunction. Nat. Commun..

[16] White E.J., Matsangos A.E., Wilson G.M. (2017). AUF1 regulation of coding and noncoding RNA. Wiley Interdiscip. Rev. RNA.

[17] Yoon J.H., De S., Srikantan S., Abdelmohsen K., Grammatikakis I., Kim J., Kim K.M., Noh J.H., White E.J., Martindale J.L. (2014). PAR-CLIP analysis uncovers AUF1 impact on target RNA fate and genome integrity. Nat. Commun..

[18] Zucconi B.E., Wilson G.M. (2011). Modulation of neoplastic gene regulatory pathways by the RNA-binding factor AUF1. Front. Biosci..

[19] Chenette D.M., Cadwallader A.B., Antwine T.L., Larkin L.C., Wang J., Olwin B.B., Schneider R.J. (2016). Targeted mRNA Decay by RNA Binding Protein AUF1 Regulates Adult Muscle Stem Cell Fate, Promoting Skeletal Muscle Integrity. Cell Rep..

[20] Lu J.Y., Sadri N., Schneider R.J. (2006). Endotoxic shock in AUF1 knockout mice mediated by failure to degrade proinflammatory cytokine mRNAs. Genes Dev..

[21] Sadri N., Schneider R.J. (2009). Auf1/Hnrnpd-deficient mice develop pruritic inflammatory skin disease. J. Investig. Dermatol..

[22] Moore A.E., Chenette D.M., Larkin L.C., Schneider R.J. (2014). Physiological networks and disease functions of RNA-binding protein AUF1. Wiley Interdiscip. Rev. RNA.

[23] Tripathi M., Yen P.M., Singh B.K. (2020). Protocol to Generate Senescent Cells from the Mouse Hepatic Cell Line AML12 to Study Hepatic Aging. STAR Protoc..

[24] Jung M., Ryu S., Kim C., Cha S., Kang H., Ji E., Hong Y., Lee Y., Han S., Jeong S.M. (2022). RNA binding protein HuD mediates the crosstalk between β cells and islet endothelial cells by the regulation of Endostatin and Serpin E1 expression. Cell Death Dis..

[25] Ryu S., Jung M., Kim C., Kang H., Han S., Cha S., Jeong S.M., Lee E.K. (2022). Loss of RNA binding protein HuD facilitates the production of the senescence-associated secretory phenotype. Cell Death Dis..

[26] Chaudhry A., Shi R., Luciani D.S. (2020). A pipeline for multidimensional confocal analysis of mitochondrial morphology, function, and dynamics in pancreatic β-cells. Am. J. Physiol. Endocrinol. Metab..

[27] Greotti E., Fortunati I., Pendin D., Ferrante C., Galla L., Zentilin L., Giacca M., Kaludercic N., Di Sante M., Mariotti L. (2019). mCerulean3-Based Cameleon Sensor to Explore Mitochondrial Ca(2+) Dynamics In Vivo. iScience.

[28] Vallese F., Catoni C., Cieri D., Barazzuol L., Ramirez O., Calore V., Bonora M., Giamogante F., Pinton P., Brini M. (2020). An expanded palette of improved SPLICS reporters detects multiple organelle contacts in vitro and in vivo. Nat. Commun..

[29] Giamogante F., Barazzuol L., Maiorca F., Poggio E., Esposito A., Masato A., Napolitano G., Vagnoni A., Cali T., Brini M. (2024). A SPLICS reporter reveals α-synuclein regulation of lysosome-mitochondria contacts which affects TFEB nuclear translocation. Nat. Commun..

[30] Schindelin J., Arganda-Carreras I., Frise E., Kaynig V., Longair M., Pietzsch T., Preibisch S., Rueden C., Saalfeld S., Schmid B. (2012). Fiji: An open-source platform for biological-image analysis. Nat. Methods.

[31] Schneider C.A., Rasband W.S., Eliceiri K.W. (2012). NIH Image to ImageJ: 25 years of image analysis. Nat. Methods.

[32] Gallage S., Gil J. (2016). Mitochondrial Dysfunction Meets Senescence. Trends Biochem. Sci..

[33] Martini H., Passos J.F. (2023). Cellular senescence: All roads lead to mitochondria. FEBS J..

[34] Dematteis G., Tapella L., Casali C., Talmon M., Tonelli E., Reano S., Ariotti A., Pessolano E., Malecka J., Chrostek G. (2024). ER-mitochondria distance is a critical parameter for efficient mitochondrial Ca(2+) uptake and oxidative metabolism. Commun. Biol..

[35] Rizzuto R., Pinton P., Carrington W., Fay F.S., Fogarty K.E., Lifshitz L.M., Tuft R.A., Pozzan T. (1998). Close contacts with the endoplasmic reticulum as determinants of mitochondrial Ca^2+^ responses. Science.

[36] Rieusset J. (2018). The role of endoplasmic reticulum-mitochondria contact sites in the control of glucose homeostasis: An update. Cell Death Dis..

[37] DeMaria C.T., Brewer G. (1996). AUF1 binding affinity to A+U-rich elements correlates with rapid mRNA degradation. J. Biol. Chem..

[38] Du T., Fang Q., Zhang Z., Zhu C., Xu R., Chen G., Wang Y. (2022). Lentinan Protects against Nonalcoholic Fatty Liver Disease by Reducing Oxidative Stress and Apoptosis via the PPARα Pathway. Metabolites.

[39] Fortier M., Cadoux M., Boussetta N., Pham S., Donne R., Couty J.P., Desdouets C., Celton-Morizur S. (2019). Hepatospecific ablation of p38α MAPK governs liver regeneration through modulation of inflammatory response to CCl(4)-induced acute injury. Sci. Rep..

[40] Nakata R., Hyodo F., Murata M., Eto H., Nakaji T., Kawano T., Narahara S., Yasukawa K., Akahoshi T., Tomikawa M. (2017). In vivo redox metabolic imaging of mitochondria assesses disease progression in non-alcoholic steatohepatitis. Sci. Rep..

[41] Munakarmi S., Gurau Y., Shrestha J., Risal P., Park H.S., Shin H.B., Jeong Y.J. (2022). Hepatoprotective Effects of a Natural Flavanol 3,3′-Diindolylmethane against CCl_4_-Induced Chronic Liver Injury in Mice and TGFβ1-Induced EMT in Mouse Hepatocytes via Activation of Nrf2 Cascade. Int. J. Mol. Sci..

[42] Shao M., Wang Y., Dong H., Wang L., Zhang X., Han X., Sang X., Bao Y., Peng M., Cao G. (2023). From liver fibrosis to hepatocarcinogenesis: Role of excessive liver H_2_O_2_ and targeting nanotherapeutics. Bioact. Mater..

[43] Mun H., Shin C.H., Kim M., Chang J.H., Yoon J.H. (2025). RNA-binding protein AUF1 suppresses cellular senescence and glycolysis by targeting PDP2 and PGAM1 mRNAs. Aging.

[44] Pont A.R., Sadri N., Hsiao S.J., Smith S., Schneider R.J. (2012). mRNA decay factor AUF1 maintains normal aging, telomere maintenance, and suppression of senescence by activation of telomerase transcription. Mol. Cell.

[45] Tsitsipatis D., Grammatikakis I., Driscoll R.K., Yang X., Abdelmohsen K., Harris S.C., Yang J.H., Herman A.B., Chang M.W., Munk R. (2021). AUF1 ligand circPCNX reduces cell proliferation by competing with p21 mRNA to increase p21 production. Nucleic Acids Res..

[46] Wang W., Martindale J.L., Yang X., Chrest F.J., Gorospe M. (2005). Increased stability of the p16 mRNA with replicative senescence. EMBO Rep..

[47] Kumar V., Kumar A., Kumar M., Lone M.R., Mishra D., Chauhan S.S. (2022). NFκB (RelA) mediates transactivation of hnRNPD in oral cancer cells. Sci. Rep..

[48] Al-Khalaf H.H., Aboussekhra A. (2014). MicroRNA-141 and microRNA-146b-5p inhibit the prometastatic mesenchymal characteristics through the RNA-binding protein AUF1 targeting the transcription factor ZEB1 and the protein kinase AKT. J. Biol. Chem..

[49] Dai X., Wang Y., Dong X., Sheng M., Wang H., Shi J., Sheng Y., Liu L., Jiang Q., Chen Y. (2020). Downregulation of miRNA-146a-5p promotes malignant transformation of mesenchymal stromal/stem cells by glioma stem-like cells. Aging.

[50] Korolchuk V.I., Miwa S., Carroll B., von Zglinicki T. (2017). Mitochondria in Cell Senescence: Is Mitophagy the Weakest Link?. EBioMedicine.

[51] Kelly G., Kataura T., Panek J., Ma G., Salmonowicz H., Davis A., Kendall H., Brookes C., Ayine-Tora D.M., Banks P. (2024). Suppressed basal mitophagy drives cellular aging phenotypes that can be reversed by a p62-targeting small molecule. Dev. Cell.

[52] Singh B.K., Tripathi M., Sandireddy R., Tikno K., Zhou J., Yen P.M. (2020). Decreased autophagy and fuel switching occur in a senescent hepatic cell model system. Aging.

