# Supplementary material for: AUF1 Restrains Hepatocyte Senescence by Maintaining Mitochondrial Homeostasis in AML12 Hepatocyte Model
Source: Cells. 2025 Dec 26;15(1):48. doi: 10.3390/cells15010048 (PMC12785964; doi:10.3390/cells15010048)
Supplement: Supplementary file 1 [file cells-15-00048-s001.zip › cells-4030920-supplementary.pdf]

Supplementary files

Table S1. Oligo sequences used in this study

siRNAs	Sense sequence (5′ → 3′)	Antisense sequence (5′ → 3′)
siControl	AAUUCUCCGAACGUGUCACGUUU	ACGUGACACGUUCGGAGAAUUU
siAUF1 #1	CCACAAAGAAAGAUCUGAAUU	UUCAGAUCUUUCUUUGUGGUU
siAUF1 #2	CCAGUAUUGUAGAGCAAGUUU	ACUUGCUCUACAAUACUGGUU
**For qPCR**	**Forward primer (5′ → 3′)**	**Reverse primer (5′ → 3′)**
Gapdh	AGGTCGGTGTGAACGGATTTG	TGTAGACCATGTAGTTGAGGTCA
AUF1	AGTCGGAGAGTGTAGATAAGGTC	GGCCCTTTTAGGATCAATGACTT
p16	GCTCAACTACGGTGCAGATTC	GCACGATGTCTTGATGTCCC
p21	CCTGGTGATGTCCGACCTG	CCATGAGCGCATCGCAATC
p53	GGCAACTATGGCTTCCACCT	TCCGTCATGTGCTGTGACTT
Opa1	CTCCCGACACAAAGGAAACTAT	AATACTGCGCTCAGCATCTAC
Mfn2	CTGCACCGCCATATAGAGGA	AGGGAGAAACACTGTCGAGG
Mff	ATGCCAGTGTGATAATGCAAGT	CTCGGCTCTCTTCGCTTTG
Drp1	TAAGCCCTGAGCCAATCCATC	CATTCCCGGTAAATCCACAAGT
**For BPD**	**Forward primer (5′ → 3′)** (T7: CCAAGCTTCTAATACGACTCACTATAGGGAGA)	**Reverse primer (5′ → 3′)**
Opa1 3U1	(T7) CGTTTGAGCAGAAGAGCCCT	ACATTTCCAAGGAAACTGGTT
Opa1 3U2	(T7) GTTTAGATTGGATTCATATCTCT	GGTTTAAAAATAATACAAAGTCC
Mfn2 3U1	(T7) CTGTGCATTTGATAAAGTTTTC	GATGAGCCAAAATTAGTGAGGT
Mfn2 3U2	(T7) GGAAAGCAGTTTTAGAGTGAT	CACCTAAATATACCAGAAGCA
GAPDH 3U	(T7) CCTCAACGACCACTTTGTCA	GGTTGAGCACAGGGTACTTTAT

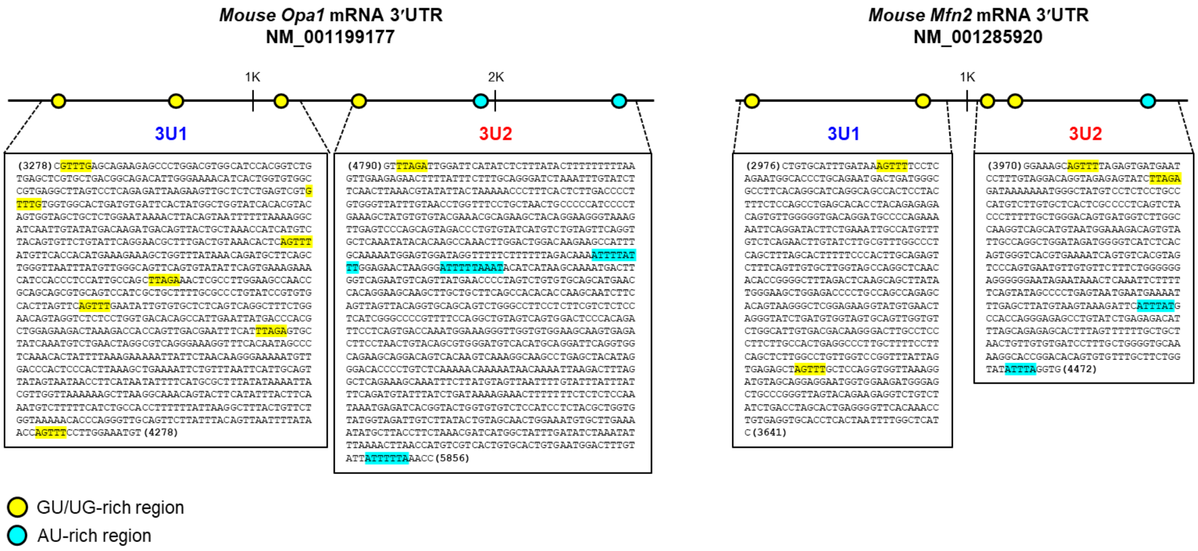

Supplementary Figure S1. Analysis of AUF1 binding sequences in the 3′UTRs of mouse *Opa1* and *Mfn2* mRNAs.

The 3′UTRs of mouse *Opa1* (NM_001199177) (A) and *Mfn2* (NM_001285920) (B) mRNAs were analyzed for potential AUF1 binding regions, based on the previously reported AUF1 binding motif [1.2]. The predicted AUF1 binding regions are highlighted in yellow (GU/ UG-rich region) and cyan (AU-rich region).

[1] Yoon, J.H.; De, S.; Srikantan, S.; Abdelmohsen, K.; Grammatikakis, I.; Kim, J.; Kim, K.M.; Noh, J.H.; White, E.J.; Martindale, J.L.; et al. PAR-CLIP analysis uncovers AUF1 impact on target RNA fate and genome integrity. Nat Commun 2014, 5, 5248, doi:10.1038/ncomms6248.

[2] DeMaria, C.T.; Brewer, G. AUF1 binding affinity to A+U-rich elements correlates with rapid mRNA degradation. J Biol Chem 1996, 271, 12179–12184, doi:10.1074/jbc.271.21.12179.
